# A New Recombinant BCG Vaccine Induces Specific Th17 and Th1 Effector Cells with Higher Protective Efficacy against Tuberculosis

**DOI:** 10.1371/journal.pone.0112848

**Published:** 2014-11-14

**Authors:** Adeliane Castro da Costa, Abadio de Oliveira Costa-Júnior, Fábio Muniz de Oliveira, Sarah Veloso Nogueira, Joseane Damaceno Rosa, Danilo Pires Resende, André Kipnis, Ana Paula Junqueira-Kipnis

**Affiliations:** 1 Laboratório de Imunopatologia das Doenças Infecciosas, Instituto de Patologia Tropical e Saúde Pública, Universidade Federal de Goiás, Goiânia, Goiás, Brazil; 2 Laboratório de Bacteriologia Molecular, Instituto de Patologia Tropical e Saúde Pública, Universidade Federal de Goiás, Goiânia, Goiás, Brazil; Colorado State University, United States of America

## Abstract

Tuberculosis (TB) is an infectious disease caused by *Mycobacterium tuberculosis* (Mtb) that is a major public health problem. The vaccine used for TB prevention is *Mycobacterium bovis* bacillus Calmette-Guérin (BCG), which provides variable efficacy in protecting against pulmonary TB among adults. Consequently, several groups have pursued the development of a new vaccine with a superior protective capacity to that of BCG. Here we constructed a new recombinant BCG (rBCG) vaccine expressing a fusion protein (CMX) composed of immune dominant epitopes from Ag85C, MPT51, and HspX and evaluated its immunogenicity and protection in a murine model of infection. The stability of the vaccine *in vivo* was maintained for up to 20 days post-vaccination. rBCG-CMX was efficiently phagocytized by peritoneal macrophages and induced nitric oxide (NO) production. Following mouse immunization, this vaccine induced a specific immune response in cells from lungs and spleen to the fusion protein and to each of the component recombinant proteins by themselves. Vaccinated mice presented higher amounts of Th1, Th17, and polyfunctional specific T cells. rBCG-CMX vaccination reduced the extension of lung lesions caused by challenge with Mtb as well as the lung bacterial load. In addition, when this vaccine was used in a prime-boost strategy together with rCMX, the lung bacterial load was lower than the result observed by BCG vaccination. This study describes the creation of a new promising vaccine for TB that we hope will be used in further studies to address its safety before proceeding to clinical trials.

## Introduction

Tuberculosis (TB) is a public health problem causing 8.6 million new cases and 1.3 million deaths annually [1]. The causative agent of TB is *Mycobacterium tuberculosis* (Mtb), an intracellular pathogen that after infecting the host can either cause active disease or remain latent. In this context, it is estimated that one third of the world population is latently infected with Mtb, of which approximately 10% will develop active disease [2], [1]. Currently, the vaccine used for TB prevention is Bacillus Calmette-Guérin (BCG), an attenuated *Mycobacterium bovis* strain used since 1921 [3]. Despite being the only approved vaccine for human use and conferring protection against tuberculous meningitis and miliary TB in children, its protective efficacy remains questionable, as it does not protect young adults against pulmonary TB [4], [5], [6].

The factors determining the variable protective mechanisms induced by BCG are not well understood. Some suppositions point towards the BCG sub-strain characteristics. It has acquired genotypic and phenotypic differences, such as residual virulence and epitope number variation, after the attenuation process and the several sub-culturing passages made through the years [7]–[8]. In addition, BCG has limited capacity to induce long lasting memory and, in humans, the vaccine induces an immune response with Th1 effector cells producing IFN-γ [9], [10], [11]. Although IFN-γ is crucial for the immune response to Mtb, studies have shown this cytokine is not a surrogate marker of the protection conferred by BCG [12], [11]. To address this matter, several groups have been working on the development of protein subunit vaccines, new adjuvants, attenuated/auxotrophic Mtb strains, and recombinant BCG (rBCG) vaccines, among other approaches [13]–[14]. Different strategies are being used by the groups modifying BCG, such as the expression of immunodominant Mtb antigens [15], the association of re-introduction and super-expression of antigens lost during the process of BCG attenuation [16], the development of rBCG expressing cytokines and Mtb proteins [17], and the heterologous expression of proteins in rBCG to induce CD8^+^ T lymphocytes [18].

While evaluating the rBCG vaccines produced in the last five years, it was observed that the selection of Mtb antigens used in the construction of the rBCG was more important for vaccine efficacy than the BCG subtypes used to make them [19]. However, comparing the BCG subtypes used to construct recombinant vaccines, sub strains BCG Tokyo and BCG Moreau presented more immune dominant epitopes than the other sub strains, and all rBCG produced using the Tokyo strain protected better than the wild type BCG [20]. Sequencing of the complete genome and an evaluation of the proteome profile of BCG Moreau were performed, but this strain was poorly used to build a TB recombinant vaccine [21]–[22]. Some studies have shown that BCG Moreau is a good carrier and efficiently induces a specific immune response to other diseases, such as pertussis, enteropathogenic *Escherichia coli*, or bladder cancer [23]–[25]. BCG Moreau has been used for more than 80 years in Brazil, attesting to its safety. This strain is currently being tested again as oral vaccine and is showing better performance than BCG Danish [26]. This prompted us to develop a recombinant TB vaccine using the BCG subtype Moreau.

Most of the time, the choice of the antigens used to develop an rBCG is based on the different phases of Mtb infection. Active and latent TB are distinct phases of the disease that can be characterized by their antigen expression, and these antigens are effective at inducing an immune response [27]. Our group and others have demonstrated that patients with active pulmonary TB and latently infected individuals respond differently to several Mtb antigens, such as antigen 85 complex proteins, MPT51 and HspX [28]–[31]. In our previous work, we developed a fusion protein (CMX) composed of the immunodominant antigens from Mtb: Ag85C, MPT51 and the entire HspX protein, [32] which are expressed in different stages of TB (active and latent phases of the disease). This construction retained the immunogenicity of the original proteins in vaccinated mice and was also specifically recognized by individuals with active TB [32]. To determine if this fusion protein could be expressed by a live vector, and consequently be used as a vaccine for TB, a recombinant *Mycobacterium smegmatis* (mc^2^) was designed to express CMX (*mc^2^-CMX*). This vaccine induced a specific immune response to CMX that culminated with protection similar to that observed following vaccination with BCG Moreau [33]. In this scenario, the fusion protein CMX was capable of adding important immunogenic properties to mycobacterium vaccine vectors, inducing an effective response to control Mtb infection in mice.

Based on the previous studies, both ours and others, we hypothesize that using BCG subtype Moreau to develop a new rBCG expressing CMX will add immunological characteristics that are missing in conventional BCG and therefore induce an specific immune response better able to control the infection by Mtb. Our data here show that the expression of CMX protein by the rBCG Moreau vaccine (rBCG-CMX) is a determining factor for inducing specific Th1 and Th17 responses, in addition to polyfunctional T cells. These responses may be responsible for the reduction in the inflammatory lung lesions induced by Mtb challenge in BALB/c mice and the reduction in the bacterial load. Moreover, prime vaccination with rBCG-CMX followed by boosting with rCMX further reduced the lung bacterial load as compared to the reduction caused by BCG Moreau.

## Materials and Methods

### Bacterial strains, growth conditions, and plasmid and vaccine preparations

The *M. bovis* BCG Moreau strain, kindly provided by the Butantan Institute, was grown in 7H9 media (Becton and Dickinson, Le pont de Claix- France) supplemented with 10 oleic acid, albumin, dextrose and catalase (OADC-Becton and Dickinson, Le pont de Claix- France), 0.5% glycerol and 0.05% Tween 80, at 37°C in a humid atmosphere and 5% CO_2_ for approximately three weeks.

The recombinant BCG strains were obtained after electroporation of BCG Moreau with one of the three expression plasmids (pLA71, pLA72, and pMIP12). These plasmids have mycobacteria and *Escherichia coli* replication origins and use the gene for kanamycin resistance as a selection marker, as described by Varaldo et al. (2004) [34]. The gene coding for the fusion CMX protein (Ag85C, MPT51, and HspX) [32] was obtained from Mtb (H37Rv) DNA and inserted in the pLA71, pLA73 and pMIP12 mycobacteria expression vectors as described by Junqueira-Kipnis et al. (2013) [33]. The employed expression plasmids enable the recombinant gene to be expressed with either the signal peptide of the β-lactamase from *M. fortuitum* (pLA71) or the entire β-lactamase protein (pLA73), or, alternatively, the protein can be highly expressed intracellularly (pMIP12). Transformants with empty plasmids were used as controls. The recombinant vaccines obtained were cultured under the same conditions as the BCG Moreau described above, with the addition of 20 µg/mL of kanamycin.

The vaccines were grown in a single lot in 7H9 supplemented with OADC, and the concentration of the lots were determined by plating serial dilutions of each vaccine onto 7H11 agar plates with or without kanamycin (20 µg/mL).

### Animals

BALB/c female mice, 4 to 8 weeks of age, from the Instituto de Patologia Tropical e Saúde Pública/UFG animal housing were maintained in micro-isolators equipped with HEPA filters for air intake and exhaustion, and provided with water and a chow diet *ad libitum*. The room temperature was kept at 20–24°C with a relative humidity of 40–70% and light/dark cycles of 12 hours. Mice were handled according to the Sociedade Brasileira de Ciência em Animais de Laboratório (SBCAL/COBEA) guidelines. The study was approved by the Ethical Committee for Animal use (CEUA: Comite de Ética no uso de animais; #229/11) of the Universidade Federal de Goiás.

### PCR and Western blotting

To confirm the presence of the CMX fusion gene (∼860 base pairs), a PCR reaction using Ag85C forward (5′ ggtctgcgggcccaggatg 3′) and HspX reverse (5′ tcagttggtggaccggatctgaatgtg 3′) primers (10 nmol of each) in the same conditions as described previously [32]. The expression of CMX (∼35 kDa) by the different vectors was assessed by Western blot, as described previously [33].

### Assessment of in vivo plasmid stability

BALB/c mice were immunized with 10^6^ colony forming units (CFU) of rBCG-pLA71-CMX or rBCG-pLA71 subcutaneously in the dorsal region. Animals were euthanized at different time points (3 mice/group/time point) and the dorsal tissue at the injection site was cut out and macerated. The homogenized tissue was plated onto 7H11 agar supplemented with OADC, 0.5% glycerol and 20 µg/mL of kanamycin. After incubation at 37°C with 5% CO_2_ for approximately 30 days, the plates were analyzed for bacterial growth and the numbers of CFU were determined. The DNA from a representative colony was extracted by boiling the entire colony, and the supernatant was submitted to PCR for detection of the CMX gene. This experiment was repeated three times.

### Mouse peritoneal macrophage preparation, culture, and infection

Peritoneal macrophages were obtained after injection of 1 mL of thioglycolate into the peritoneal cavity of BALB/c mice four days prior to macrophage collection. Mice were euthanized by cervical dislocation and 5 mL of ice cold phosphate buffered saline (PBS) was injected into the peritoneal cavity, followed by vigorous massage. The recovered cells were distributed in a 24 well plate at a concentration of 1×10^6^ cells per mL and incubated with 5% CO_2_ for 24 hours to allow for adherence. In some of the cultured wells, circular glass cover slides were introduced to allow for microscopic evaluation. Macrophages were infected with BCG or rBCG-CMX at a multiplicity of infection (MOI) = 10 or incubated with LPS (5 µg/mL), as a control. Infected macrophages were incubated at 37°C with 5% CO_2_. After 3 hours, the supernatant was discarded, the cells were washed, and new media was added to the wells. After 18 hours, the supernatant was collected and plated on 7H11 agar plates to determine the number of bacteria that were not phagocytosed. Infected macrophages were washed three times with RPMI medium (HIMEDIA, Mumbai-India) and then lysed with water and plated on 7H11 agar to determine the level of CFU of the intracellular bacteria. Some of the infected macrophages were kept for an additional incubation of 48 hours and used for nitric oxide (NO) quantification of the supernatant by the Griess method, as described below. The cover slides from macrophages infected for 3 hours were washed three times with PBS at 37°C, fixed with methanol and stained with Ziehl Neelsen, for acid fast bacilli visualization, or Instant Prov (Newprov, Pinhais- Brazil), for cell visualization.

### Nitric oxide determination

Supernatants (100 µL) from macrophage cultures that had been stimulated or not (control) with BCG, rBCG, or LPS were stored in a 96 well plate at –20°C until use. Fifty microliters of the supernatant was transferred to another 96 well plate and 50 µL Griess reagent (1% sulphanilamide, 2% phosphoric acid, and 0.1% naphthylethylene diamine dihydrochloride) was added, followed by 15 minutes of incubation at room temperature, protected from the light. A serial dilution of nitrite was included in additional wells to provide a standard curve for comparison. The absorbance was measured in a spectrophotometer (Thermo LabSystems Multiskan RC/MS/EX Microplate Reader) at 595 nm.

### BCG and rBCG-CMX immunizations

BALB/c mice were separated into three groups: Control, BCG Moreau, and rBCG-CMX. Five to six animals were used in each group. Prior to use, the vaccines were thawed and the concentrations adjusted with PBS/0.05% Tween 80, so that each animal would receive 10^6^ CFU in 100 µL by subcutaneous injection in the dorsal region. The vaccine concentrations were confirmed by plating the remaining inocula on 7H11 agar supplemented with OADC. An additional group of animals, previously vaccinated with rBCG-CMX (N = 5) was given a booster, 30 days later with 20 µg/mL of rCMX/CPG DNA prepared as described at de Souza et al, 2012 [32]. This experiment was repeated two times in BALB/c mice and one time in C57BL/6 mice.

### Cell preparation for immune response evaluation

Thirty days after immunization, six animals from each group of the BALB/c mice were euthanized and the spleens and left lung lobes were collected. Spleens were prepared into single cell suspensions using 70 µm cell strainers (BD Biosciences, Lincoln Park, NJ) and the cells were resuspended with RPMI medium. Erythrocytes were lysed with lysis solution (0.15 M NH_4_Cl, 10 mM KHCO_3_) and the cells were washed and resuspended with RPMI supplemented with 20% fetal calf serum, 0.15% sodium bicarbonate, 1% L-glutamine (200 mM; Sigma-Aldrich-Brazil, São Paulo), 1% non-essential amino acids (Sigma-Aldrich). Cells were counted in a Neubauer chamber and the concentration was adjusted to 1×10^6^ cells/mL. Prior to collection, the lungs were perfused with ice-cold PBS containing 45 U/mL of heparin (Sigma-Aldrich-Brazil, São Paulo) and processed as described previously [33]. The lungs were digested with DNAse IV (30 µg/mL; Sigma-Aldrich) and collagenase III (0.7 mg/mL; Sigma-Aldrich-Brazil, São Paulo) for 30 min at 37°C. The digested lungs were prepared into single cell suspensions using 70 µm cell strainers and submitted to erythrocyte lysis. The cells were washed and resuspended with RPMI, and the concentrations were adjusted to 1×10^6^ cells/mL.

### Ag85 (Rv0129c), MPT51 (Rv3803c), HspX (Rv2031c) and CMX specific cytokine evaluation by lung and spleen lymphocytes

In a 96 well cell culture plate (CellWells TM), 200 µL of spleen or lung cell suspensions were cultivated without (media alone) or with recombinant CMX or with only one of the component recombinant proteins, Ag85, MPT51 or HspX (single proteins were used at a concentration of 10 µg/mL) or ConA (positive control) in a 5% CO_2_ incubator at 37°C for 4 hours. Monensin (3 µM; eBioscience) was then added to the wells and the cultures were further incubated for 4 hours. Cells were treated with 0.1% sodium azide in PBS for 30 min at room temperature. After centrifuging, the cells were stained with anti-CD4 Percp (eBioscience, clone RM4-5) or anti-CD4 FITC (BD PharMingen, clone RM4-5) for 30 min. Cells were then, permeabilized with Perm Fix/Perm Wash (BD PharMingen), washed with 0.1% sodium azide in PBS, and then stained with the following antibodies to access the expressions of a panel of Th1 cytokines: anti-TNF-α FITC (BD PharMingen-MP6; clone: XT22), anti-IL-2 PE (eBioscience-JES6; clone: 5H4), and anti-IFN-γ APC (eBioscience; clone: XMG1.2). To access the expression of a panel of Th17 cytokines, cells were stained with: anti-IL-2 PE (eBioscience, clone: JES6-5H4), anti-IL-17A Percp (eBioscience, clone: eBio17B7), and anti-IFN-γ APC (eBioscience, clone: XMG1.2) for 30 min. Cell acquisition of 100,000 events per sample was performed in a BD FACS Verse (Universidade de Brasília-UNB) flow cytometer and the acquired data were analyzed using FlowJo 8.7 software. Lymphocytes were selected based on their size (Forward scatter, FSC) and granularity (side scatter, SSC). The specific immune responses were determined by subtracting the result of the media alone stimulation from the responses to each of the antigens.

### *Mycobacterium tuberculosis* intravenous infection

The *Mycobaterium tuberculosis* (H37Rv) strain was maintained as described previously [33]. A vial from a constant lot was thawed and the inoculum was adjusted to the concentration of 10^6^ CFU/mL by diluting with PBS containing 0.05% Tween 80. Ninety days after immunization with rBCG-CMX or BCG Moreau, 100 µL of the inoculum was injected into the retro-orbital plexus. The bacterial load of infection was determined by plating the lung homogenates from one mouse from each group on the day following infection on 7H11 agar supplemented with OADC. Forty-five days after infection, mice were euthanized and the anterior and mediastinal right lung lobes were collected, homogenized, and plated on 7H11 agar supplemented with OADC. The bacterial load was determined by counting the CFU after 21 days of incubation at 37°C.

### Histopathology

The lungs of mice euthanized 45 days after the Mtb challenge were perfused with 0.05% heparin by injection in the heart right ventricle. The posterior right lobes were collected, conditioned in histological cassettes, and fixed with 10% buffered formaldehyde. Samples were sectioned into 5 µm thick slices and stained with hematoxylin and eosin (HE) for analysis via microscopy (Axio scope.A1 - Carl Zeiss). Scores for the observed lesions were determined based on the area with lesions relative to the area of the total visual field. The results are presented as the percentage of area with lesions. Three different fields were evaluated per slide for each animal of each group.

### Statistical analysis

The data were analyzed using Microsoft Office Excel 2011 and Prism (version 5.0c, GraphPad) software. The results represent the mean and standard deviation for each experimental group. The results from rBCG-CMX and BCG groups were compared using One-Way Anova followed by Dunnett’s post-hoc test. Values of p<0.05 were considered statistically significant. All experiments repetition showed similar responses.

## Results

### 1. Recombinant vaccine construction, rCMX expression analysis and *in* *vivo* plasmid stability

**Figure 1:**
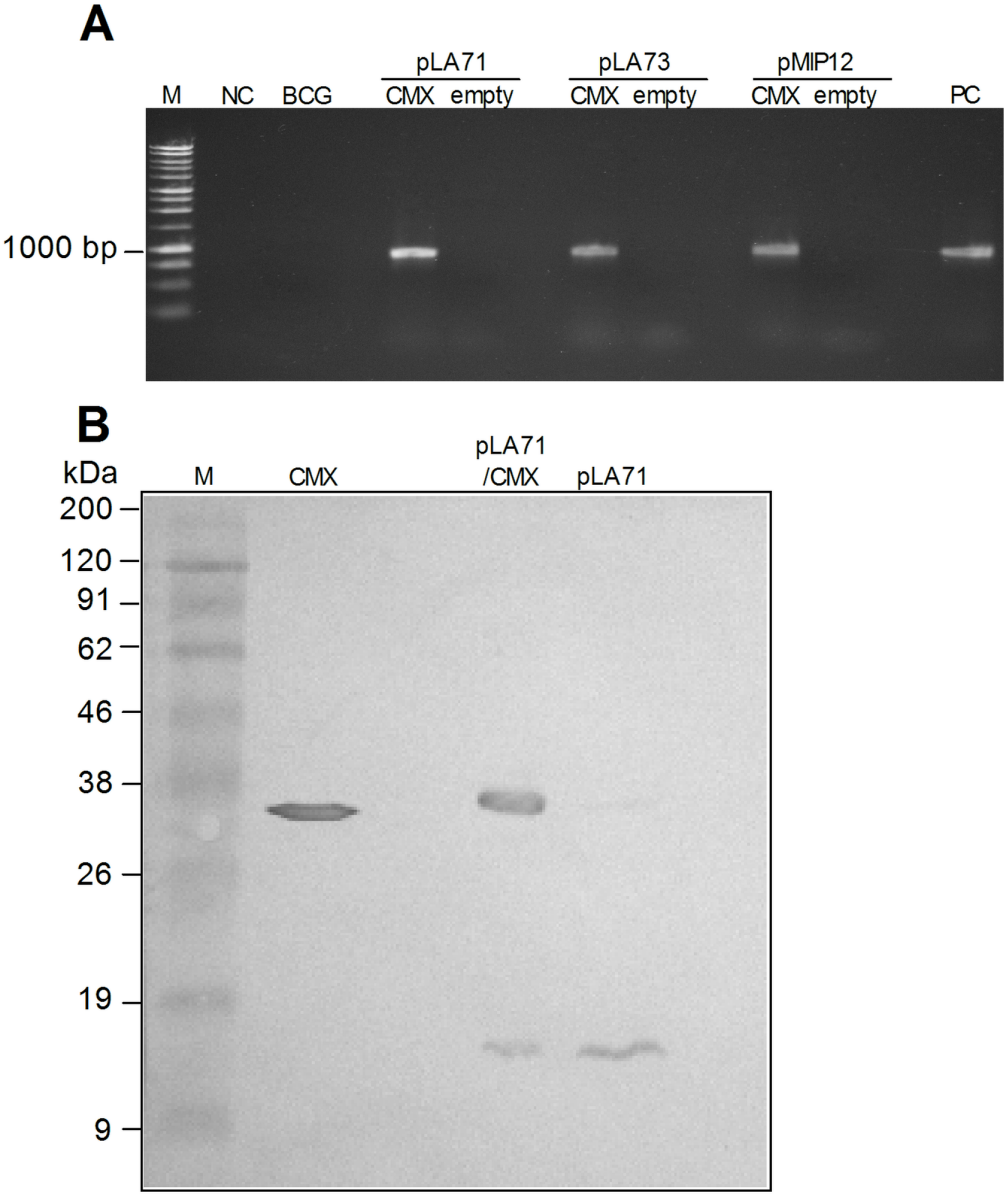
Plasmid construction and CMX expression for three different rBCG-CMX vaccines. (A) PCR products corresponding to the CMX fusion gene, CMX (∼860 bp), from all three plasmid constructions and their respective empty controls: pLA71, pLA73 and pMIP12. M: molecular weight marker; NC: negative control reaction, using water; BCG: DNA from BCG-Moreau; rBCG transformed with pLA71, pLA73 and pMIP12, with (CMX) or without (empty) the fusion gene; PC: positive control reaction. (B) Analysis of CMX expression in rBCG-pLA71/CMX. Western blot of BCG transformants containing pLA71/CMX or empty vector using polyclonal antibody produced against rCMX. M: molecular mass marker; CMX: purified recombinant CMX; pLA71/CMX: rBCG with plasmid pLA71/CMX; pLA71: rBCG with plasmid pLA71.

The expression of heterologous proteins in mycobacteria can be influenced by several factors such as administration dose, cellular localization, and expression stability, among others [34]–[35]. To obtain the best possible expression, we tested three different plasmid constructions to express CMX: pLA71/CMX, pLA73/CMX, and pMIP12/CMX. As shown in Figure 1A, all three constructions contained the CMX fused gene and were successfully transformed into BCG Moreau. Western blot analysis of recombinant BCG cultures revealed that only the plasmid pLA71/CMX was capable of inducing the expression of CMX protein (Fig. 1B, Figure S1). Thus we performed the following analysis only with the recombinant vaccine rBCG-pLA71/CMX, henceforward referred to as rBCG-CMX.

**Figure 2:**
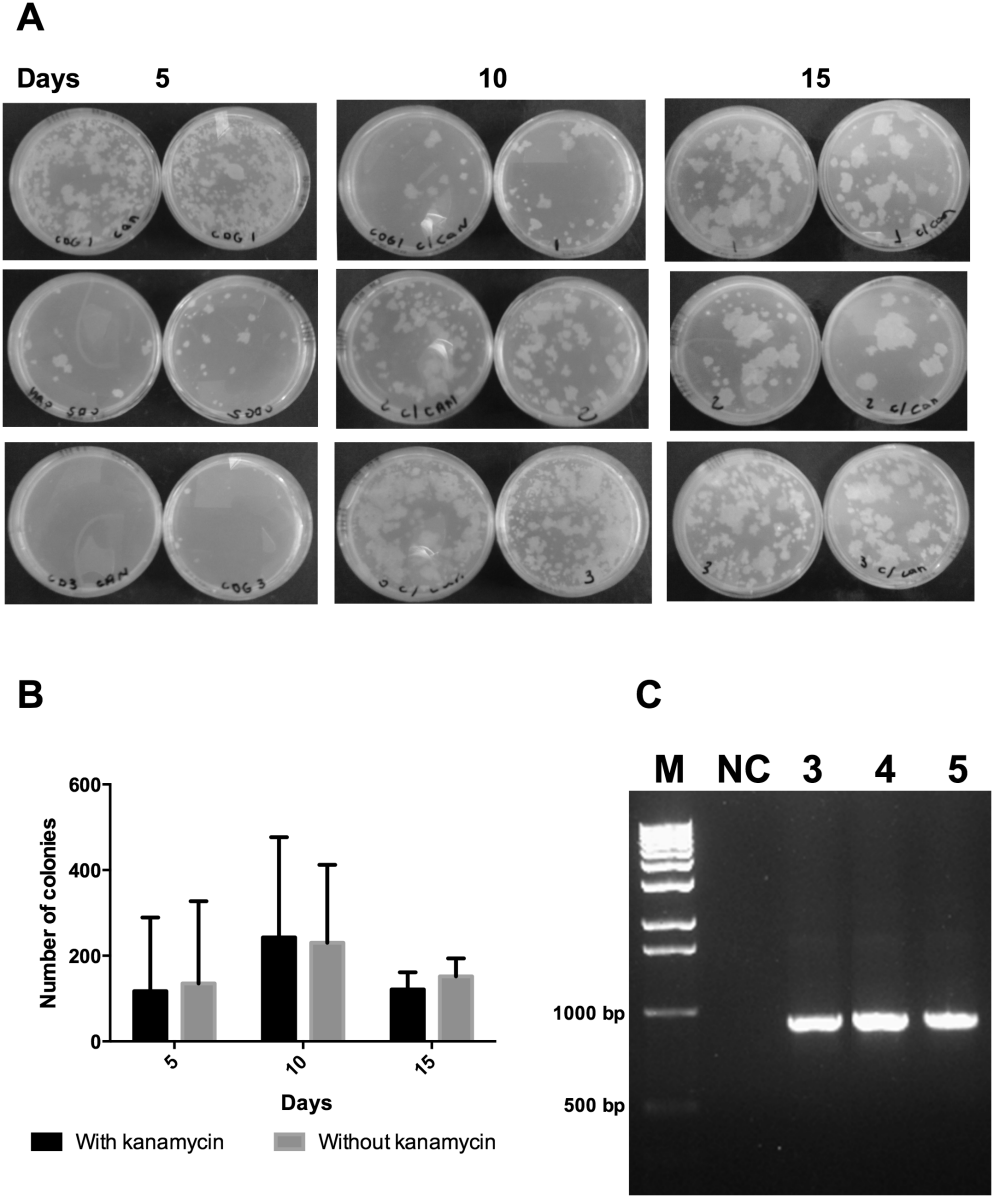
Stability of rBCG-CMX *in vivo*. (A) Images of plates showing the mycobacterial growth of rBCG-CMX recovered from the dorsal region of mice 5, 10 and 15 days after subcutaneous immunization, and plated on media with kanamycin (kan) or without (W/o). (B) CFU counts recovered at different time points from the dorsal region of mice after immunization. (C) CMX gene detection by PCR for three isolated colonies from plates W/o kanamycin (Lanes 3–5). Lanes 1: M: molecular weight marker; 2: Negative control: NC: water.

In order to verify the stability of the plasmid within the recombinant vaccine rBCG-CMX *in* *vivo* without antibiotic selective pressure, mice were vaccinated subcutaneously and the tissue of the site of infection was macerated at different time points and plated on media with or without the selective antibiotic kanamycin. As shown in Figure 2, the number of CFU recovered from media with or without antibiotic was similar, indicating that the recombinant vaccine recovered from mice retained the plasmids up to 15 days after immunization (Figs. 2A and B). The presence of plasmid was further confirmed by performing PCR specific to the CMX gene (Fig. 2C).

### 2. rBCG-CMX is phagocytosed by peritoneal macrophages at higher levels than BCG Moreau but induces similar levels of NO

**Figure 3:**
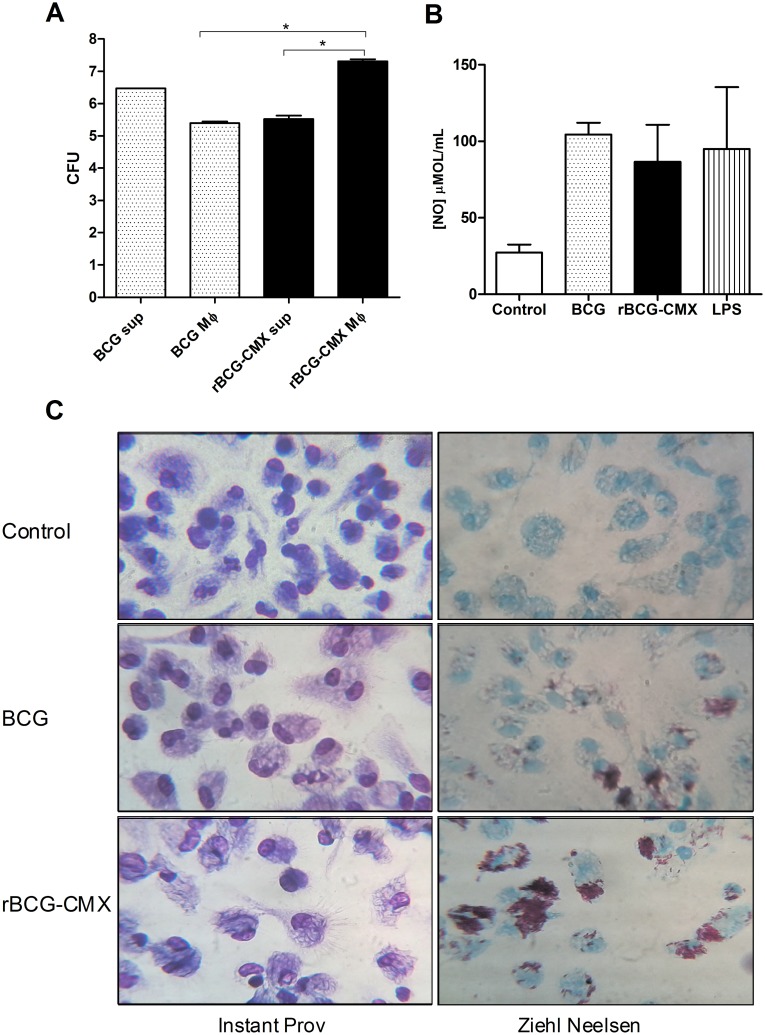
Levels of phagocytosis by peritoneal macrophages of BCG and rBCG-CMX after infection (MOI = 10). (A) Macrophages were infected with BCG or rBCG-CMX and the bacterial load in both the supernatant (sup) and inside the macrophages (Mφ) were determined. The amount of viable bacteria was determined by plating supernatant or cell lysates onto 7H11 agar supplemented with OADC and counting the CFU 28 days after incubation at 37°C. *(p<0.01) significant difference between the compared groups (log10 scale). (B) Nitric oxide (NO) production by macrophages infected with BCG or rBCG-CMX was determined. Uninfected media (Control) and LPS-stimulated (LPS) macrophages were included as controls. (C) Microscopic evaluation of peritoneal macrophages, 3 hours after infection with BCG or rBCG-CMX stained with Instant Prov or Ziehl Neelsen. Uninfected macrophages (Control) were included as a negative control. The results shown are representative of three different experiments.

Vaccine phagocytosis and processing to present antigens has been shown to be an important factor responsible for the capacity to induce a protective immune response [36]. Thus the tendency of peritoneal macrophages to phagocytose rBCG-CMX was analyzed (Fig. 3). After 18 hours of infection, the recovered CFU from rBCG-CMX infected macrophages was higher than that from BCG Moreau infected macrophages (Fig. 3A, p<0.01). Acid fast staining of infected macrophage cultures confirmed that peritoneal macrophages had higher numbers of rBCG-CMX than of BCG (Fig. 3C). The analysis of CFU from culture supernatants confirmed that the groups were equally infected by the different vaccines (Fig. 3A).

Phagocytosis induces respiratory burst activation with its consequent production of NO. As another way to evaluate phagocytosis rates, the production of nitric oxide (NO) was evaluated in the culture supernatant of infected peritoneal macrophages. No difference in NO production was observed between the two vaccines evaluated (Fig. 3B). Despite the increased number of bacilli inside the peritoneal macrophages infected with rBCG-CMX, similar levels of NO were induced by both vaccines.

### 3. rBCG-CMX vaccine induces a specific cellular immune response

**Figure 4:**
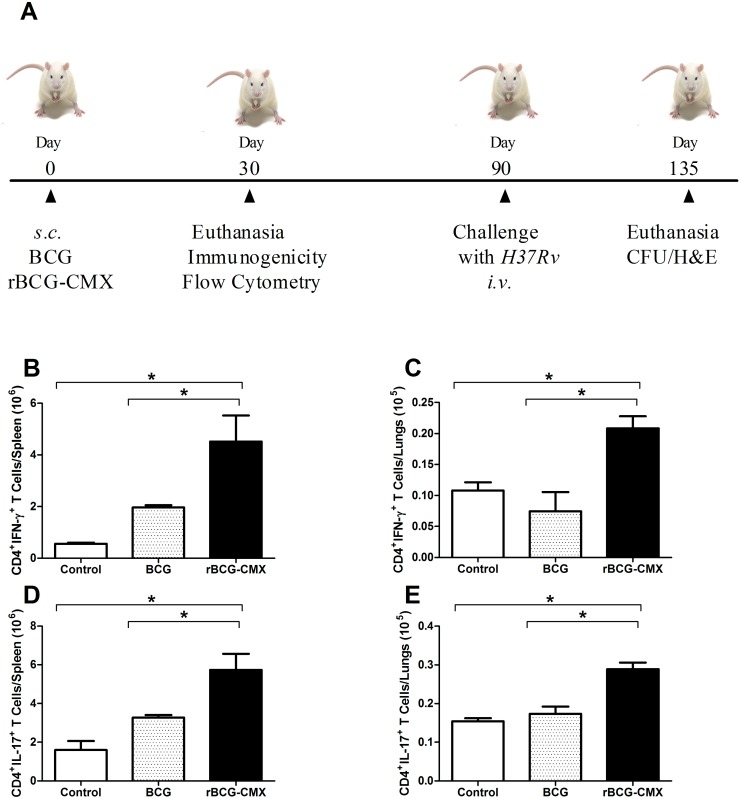
Immunogenicity of rBCG-CMX in BALB/c mice. (A) Experimental time line. BALB/c mice were immunized with rBCG-CMX or BCG Moreau. Thirty days later, 6 mice per group were euthanized for evaluation of vaccine-induced immunogenicity. Ninety days after immunization, mice were intravenously (*i.v.*) challenged with 10^5^ CFU of H37Rv. Forty-five days after *i.v.* challenge, the lung bacterial load (CFU) and lesions (H&E) were assessed. (B–E) Specific cellular immune responses induced with rCMX stimulation *ex* *vivo*. Spleen (B and D) and lung (C and E) cell suspensions from vaccinated and unvaccinated (Control) mice were stimulated with rCMX. Cells positive for both CD4 and IFN-γ (B and C) or CD4 and IL-17 (D and E) were determined by flow cytometry. Lymphocytes were selected based on size and granularity. Flow cytometry gates were set to analyze CD4^+^ T cells, and then the fluorescence of antibodies detecting IFN-γ^+^ or IL-17^+^ cells was recorded. These data are representative of two independent experiments (N = 6, *p<0.05).

Since rBCG-CMX was stable *in* *vivo* and phagocytosis of it induced macrophage activation (as measured by NO production), we questioned whether this vaccine would be able to induce a specific response to CMX and/or to the recombinant antigens alone (Fig. 4A). Immunization with rBCG-CMX vaccine induced higher numbers of CD4^+^ T lymphocytes positive for IFN-γ specific for CMX in cells from the spleen and lungs of BALB/c immunized mice 30 days after vaccination than did immunization with BCG Moreau (Figs. 4B and C, p<0.05; Th1 representative dot plots in Figure S2A). Similarly, rBCG-CMX induced higher levels of specific Th17 cells, an important group of cells for protection from Mtb and development of memory, in the cells from the spleen and lungs of immunized mice (Figs. 4D and E, p<0.05; Th17 representative dot plots in Fig. S2B).

**Figure 5:**
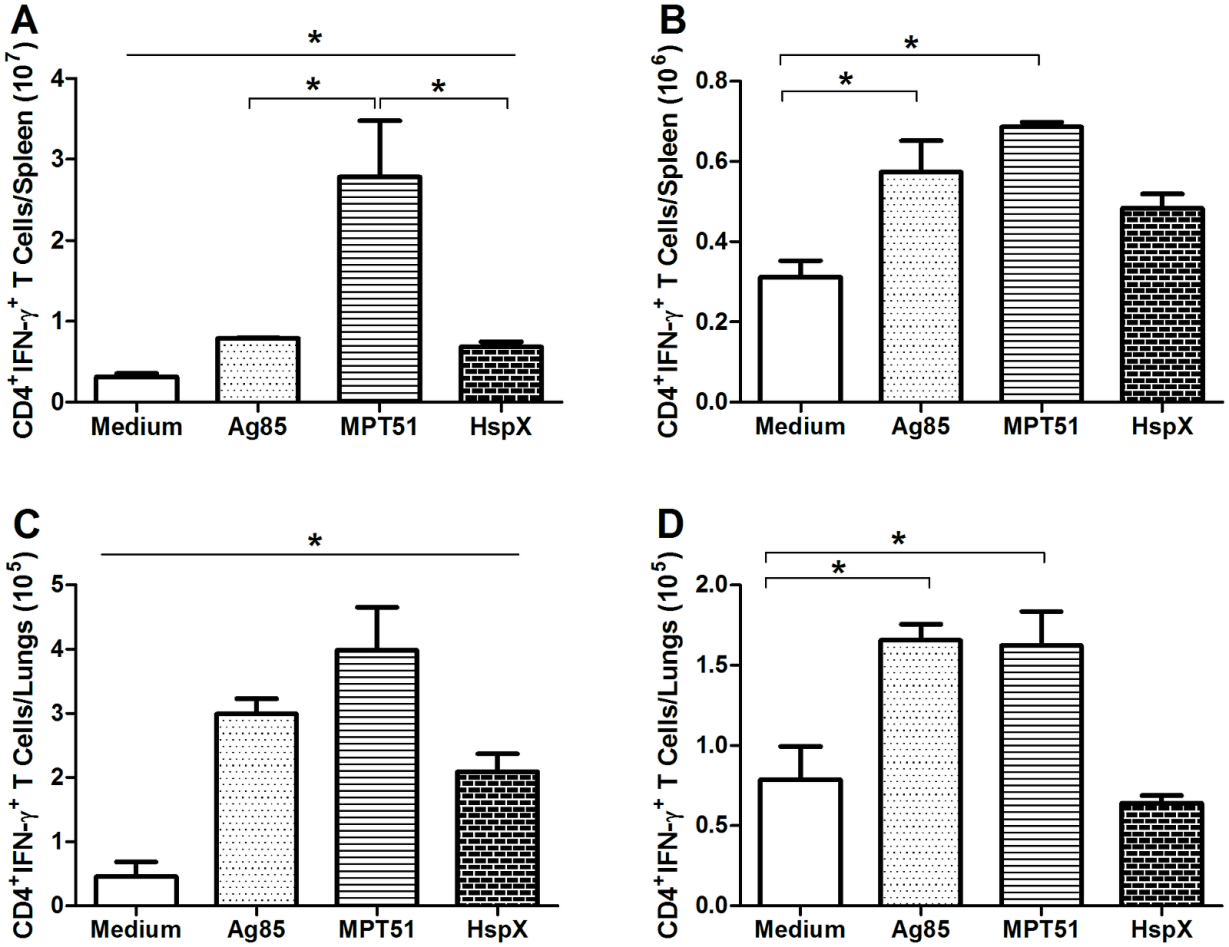
Levels of CD4^+^IFN-γ^+^ T cells induced by *ex vivo* stimulation with recombinant Ag85, MPT51, and HspX. Thirty days after vaccination, lung and spleen suspensions were stimulated *ex* *vivo* with Ag85, MPT51, HspX, or medium alone. The number of cells positive for CD4 and IFN-γ was determined by flow cytometry. Lymphocytes were selected based on size and granularity. Gates were set to analyze CD4^+^ T cells, and then the fluorescence of antibodies detecting IFN-γ^+^ cells was recorded. (A–B) Spleen cells from mice vaccinated with (A) rBCG-CMX or (B) BCG. (C–D) Lung cells from mice vaccinated with (C) rBCG-CMX or (D) BCG. In A and C, all results were different from the medium stimulation. These data are representative of two independent experiments (N = 6, *p<0.05).

**Figure 6:**
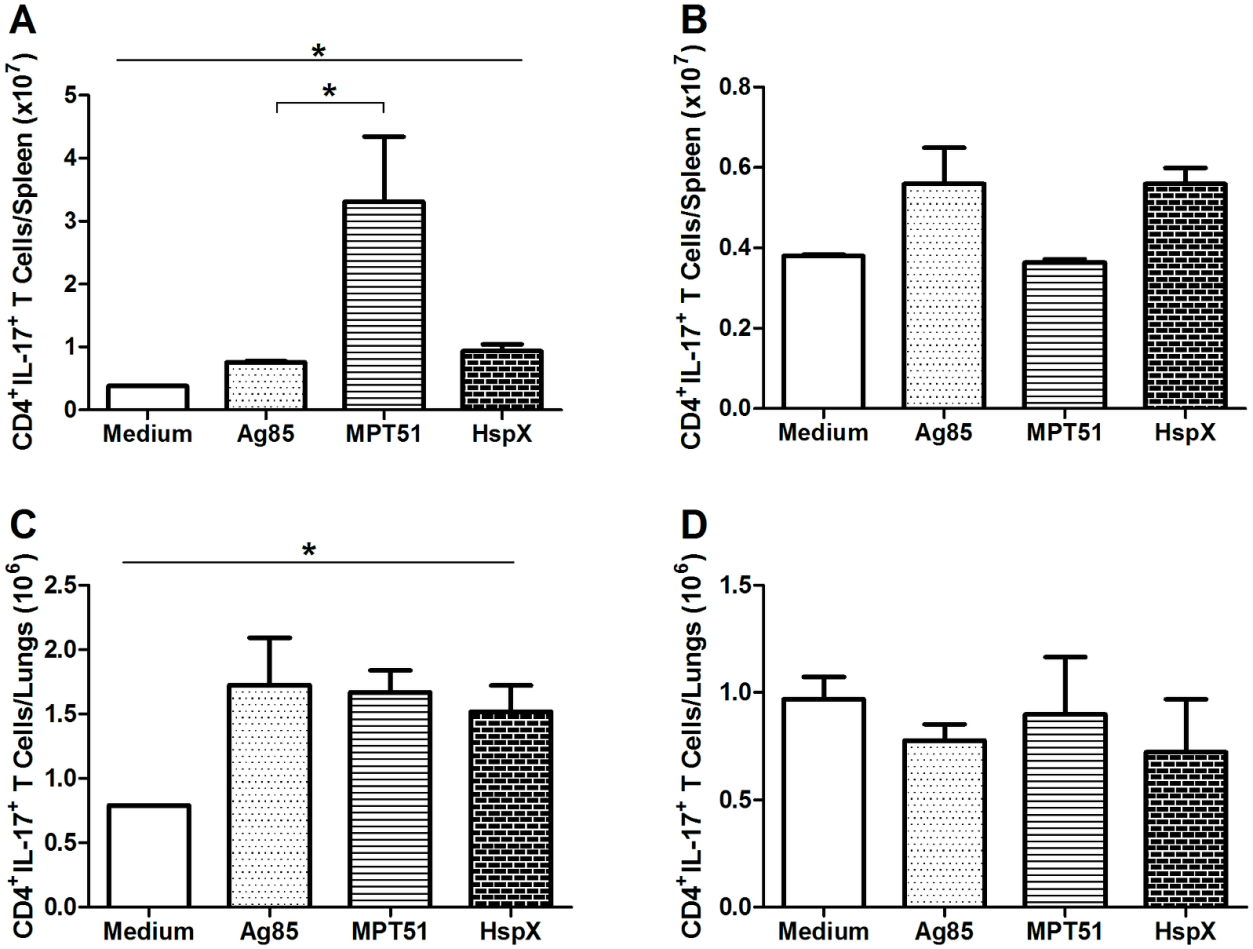
Levels of CD4^+^IL-17^+^ T cells induced by *ex vivo* stimulation with recombinant Ag85, MPT51, and HspX. Thirty days after vaccination, lung and spleen suspensions were stimulated *ex* *vivo* with Ag85, MPT51, HspX, or medium alone. The number of cells positive for CD4 and IL-17 was determined by flow cytometry. Lymphocytes were selected based on size and granularity. Gates were set to analyze CD4^+^ T cells, and then the fluorescence of antibodies detecting IL-17^+^ cells was recorded. (A–B) Spleen cells from mice vaccinated with (A) rBCG-CMX or (B) BCG. (C–D) Lung cells from mice vaccinated with (C) rBCG-CMX or (D) BCG. In A and C, all results were different from the medium stimulation. These data are representative of two independent experiments (N = 6, *p<0.05).

We next questioned which protein(s) of the recombinant CMX fusion protein could contribute to the induction of IFN-γ (Fig. 5) and/or IL-17 (Fig. 6) by CD4^+^ T lymphocytes. As depicted in Figure 5, *ex* *vivo* stimulation of spleen and lung cells from rBCG-CMX vaccinated mice with Ag85, MPT51, or HspX all specifically induced CD4^+^IFN-γ^+^ cells (Figs. 5A and C, p<0.05). A significantly higher number of spleen cells were observed responding to MPT51 than to Ag85 or HspX. In mice vaccinated with BCG, cells that were CD4^+^IFN-γ^+^ were only induced in response to Ag85 and MPT51 stimulation, but not in response to HspX. Additionally, these CD4^+^IFN-γ^+^ cells were induced to a lesser extent than in the spleen or lung cells from mice vaccinated with rBCG-CMX (Figs. 5B and D, p<0.05).

Upon antigen stimulation in cells from rBCG-CMX-immunized mice, high numbers of Th17 lymphocytes were induced. In spleen cells, the highest response was to MPT51 (Fig. 6A), while in the lungs, all antigens stimulated the production of IL-17 to a similar degree (Fig. 6C). The number of CD4^+^IL-17^+^ spleen cells responding to MPT51 was significantly higher than the amount of cells responding to Ag85 antigen (Fig. 6A, p<0.05). Th17 expression was not induced in spleen or lung cells from mice vaccinated with BCG when stimulated with any of the recombinant proteins (Ag85, MPT51 and HspX) (Figs. 6B and D).

**Figure 7:**
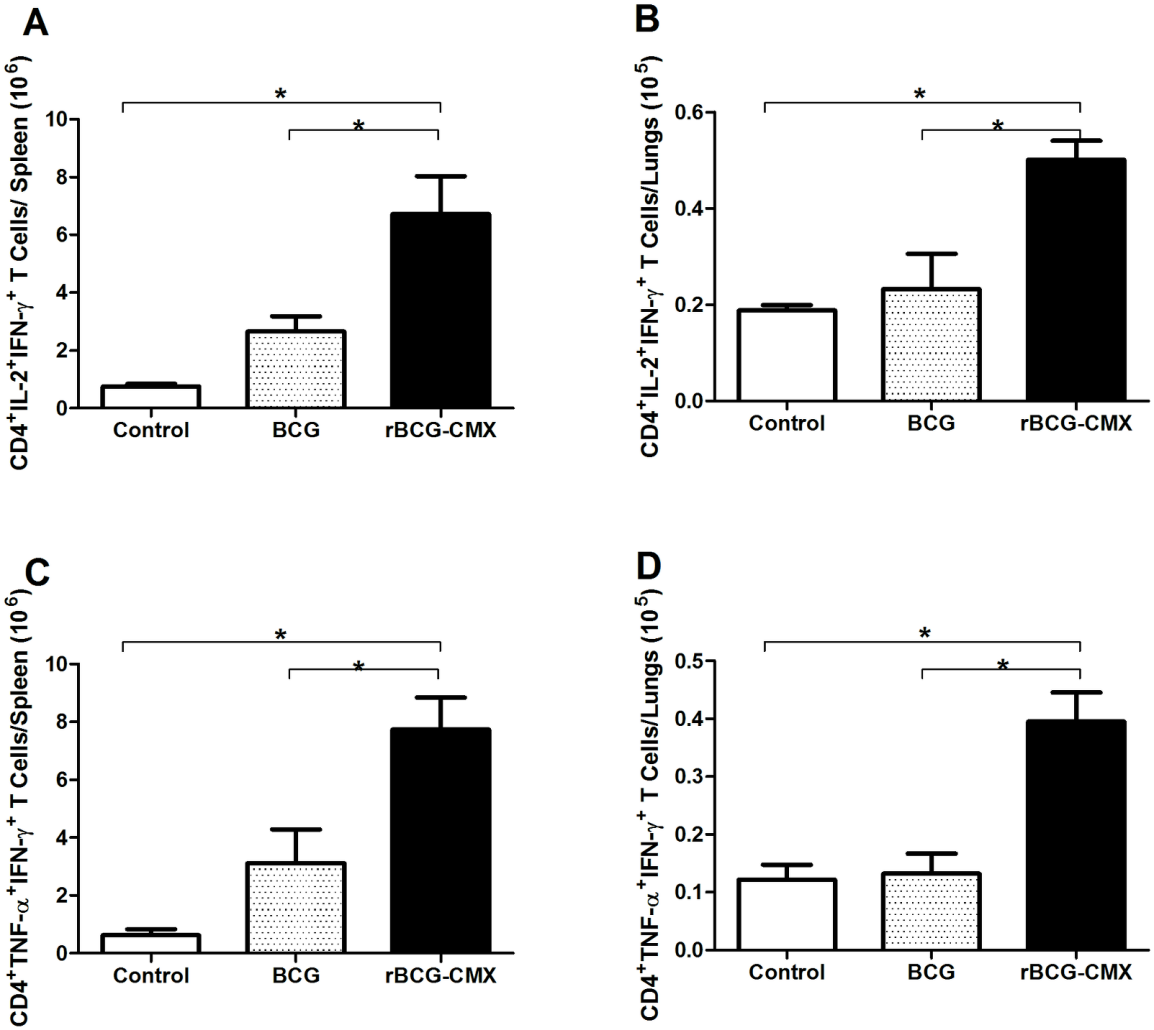
Levels of polyfunctional CD4^+^ T cells induced by BCG and rBCG-CMX vaccines. Spleen (A and C) and lung (B and D) cell suspensions from vaccinated and control mice stimulated with rCMX. (A–B) CD4^+^IL-2^+^IFN-γ^+^ cells or (C–D) CD4^+^TNF-α^+^IFN-γ^+^ cells were analyzed by flow cytometry. Lymphocytes were selected based on size and granularity. Gates were set to analyze CD4^+^ T cells, and then the fluorescence of antibodies detecting IL-2^+^ and IFN-γ^+^ or TNF-α^+^ and IFN-γ^+^ cells was recorded. These data are representative of two independent experiments (N = 6, *p<0.05).

Although the above experiments determined that the rBCG-CMX vaccine generates Th1 (IFN-γ) and Th17 specific responses, it remained important to verify the induction of polyfunctional CD4^+^ T cells, since several publications have associated these cells with protection against Mtb [33], [37]. *Ex vivo* stimulation of spleen and lung cells with CMX increased the numbers of CD4^+^ T cells positive for both IL-2 and IFN-γ (Figs. 7A and C) as well as for both TNF-α and IFN-γ (Figs. 7B and D) in cells from rBCG-CMX vaccinated mice as compared to the levels in cells from BCG Moreau vaccinated mice.

### 4. rBCG-CMX reduces the lung bacterial load

**Figure 8:**
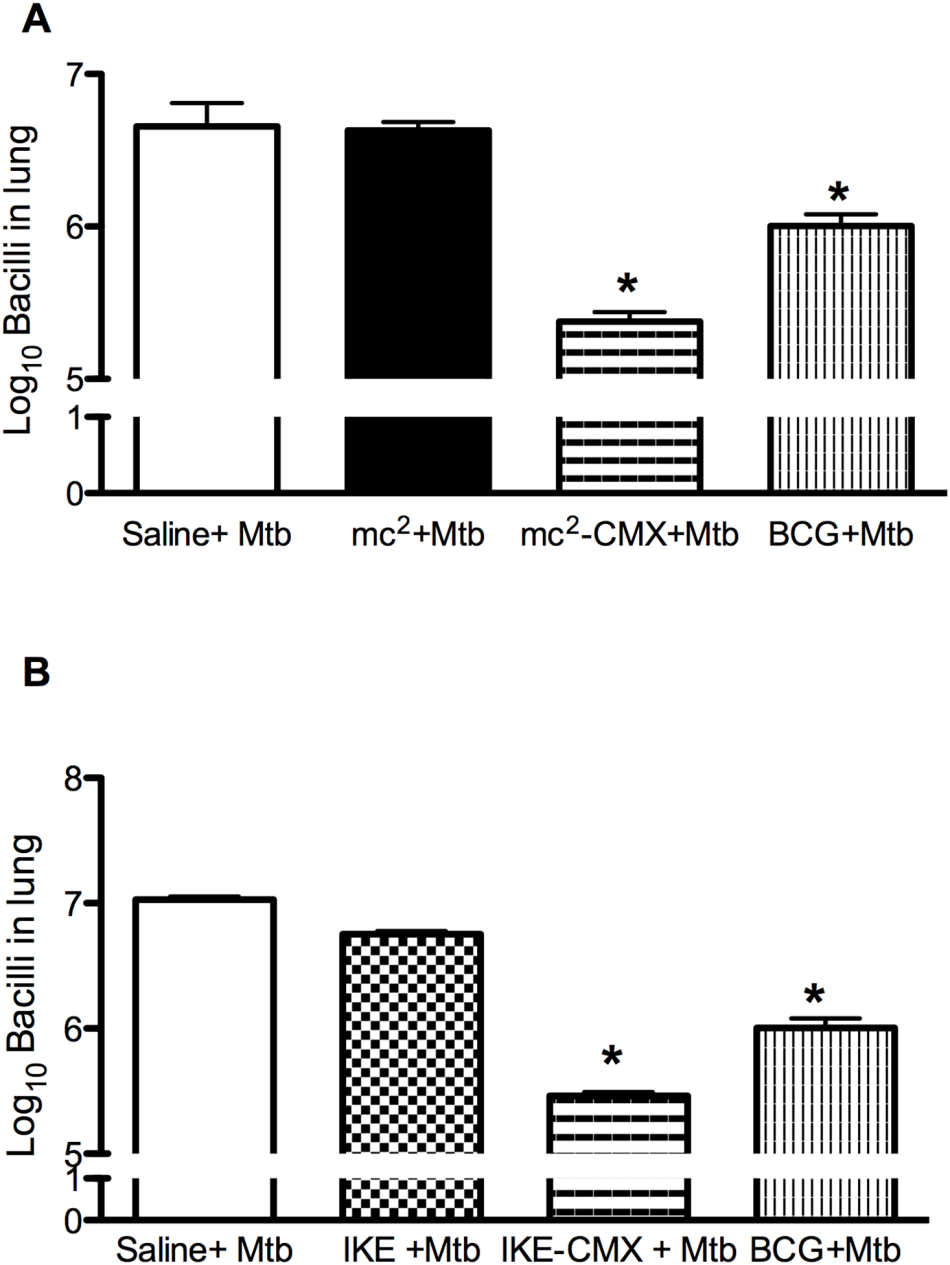
Bacterial load in the lungs of BALB/c mice 45 days after *Mycobacterium tuberculosis* challenge. Ninety days after immunization, three mice from each group (control, BCG and rBCG-CMX) were challenged with 10^5^ CFU of *Mycobacterium tuberculosis* H37Rv intravenously into the orbital sinus plexus. One additional group of animals received a booster of rCMX/CPG DNA, 30 days after rBCG-CMX vaccination and challenged with Mtb 30 days post the immunization (rBCG-CMX+CMX). Forty-five days after challenge, mice were euthanized and the anterior and mediastinal right lung lobes were collected, homogenized, and plated on Middlebrook 7H11agar supplemented with OADC to determine the bacterial load by counting the number of CFU. * Significant differences between infected (control) and vaccinated groups. # Significant differences between rBCG-CMX and rBCG-CMX+CMX groups. | Significant differences between rBCG-CMX and BCG groups analyzed by *t* test (p<0.05).

Although we found that immunization with rBCG-CMX was capable of inducing a specific immune response to CMX in BALB/c mice, this response alone is not sufficient to predict the protection properties of a vaccine. Thus, immunized mice were challenged with Mtb and the protective capacity was evaluated by assessing the bacterial load 45 days later. As observed in Figure 8, mice immunized with rBCG-CMX had a significantly lower bacterial load in the lungs than the unimmunized mice. To test if the protection could be improved in a prime-boost strategy, rBCG-CMX immunized mice were boosted 30 days later with rCMX/CPG DNA vaccine formulation and challenged with Mtb. Surprisingly, a boost with rCMX subunit vaccine showed the lowest lung bacterial load at 45 days post Mtb infection (Fig. 8).

### 5. The immune response induced by the rBCG-CMX vaccine reduces TB pulmonary lesions

**Figure 9:**
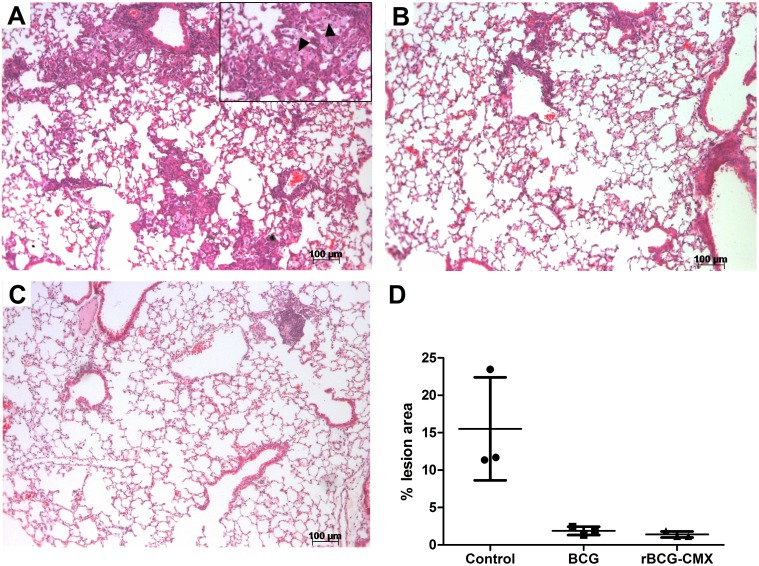
Representative lung pathology of Balb/c mice after challenge. Vaccinated mice were challenged *i.v*. with 10^5^ CFU of virulent *M. tuberculosis* H37Rv strain. Forty-five days after infection, lung tissue sections from different vaccine groups were harvested. Images are representative of two distinct experiments. HE staining is shown with 20X magnification. (A) Unvaccinated group. Black arrowheads: Foamy macrophages. (B) BCG-vaccinated group. (C) rBCG-CMX vaccinated group. (D) Histological score of the lesion area from three representative fields obtained by AxioVision 4.9.1 software, through ratio of lesioned and total field area. Data are presented as percentages (%).

Lung architecture preservation is yet another important aspect of a successful vaccine against TB. Histological analysis of the lungs of vaccinated mice challenged with Mtb showed that 45 days after challenge, unimmunized mice had intensive lymphocytic and neutrophilic infiltrates, significantly compromising the lung tissue architecture, together with the presence of a few hemorrhagic foci and foamy macrophages (Fig. 9A). BCG-vaccinated mice, instead, showed significantly fewer lung lesions, with a preservation of alveolar spaces and very limited lymphocytic infiltrate foci (Fig. 9B). The recombinant vaccine greatly preserved the lung architecture, showing very few inflammatory infiltrates (Fig. 9C). Similar results were obtained for animals immunized with rBCG-CMX and boosted with rCMX (Data not shown). The differences in inflammatory responses upon Mtb challenge between all three groups are summarized in the scores of their lung lesions, which are presented in Figure 9D.

## Discussion

In this study, a recombinant vaccine expressing the fusion protein CMX (rBCG-CMX) was used to immunize BALB/c mice and was shown to be efficient in protecting mice against Mtb challenge. The recombinant vaccine induced higher levels of CD4^+^IFN-γ^+^ and CD4^+^IL-17^+^ T cells, as well as higher levels of CD4^+^TNF-α^+^IFN-γ^+^ and CD4^+^IL-2^+^IFN-γ^+^ polyfunctional T lymphocytes specific for CMX in BALB/c mice.

During the attenuation process of BCG, some antigens important for the induction of a protective immune response were lost [38]. This is thought to be one reason that BCG does not provide long lasting protection in humans. In the pursuit of a new TB vaccine, several groups have tried to insert heterologous genes into BCG and in doing so many different expression systems have been tested [35]. Our approach was to test three different plasmid constructions to express the fusion protein CMX. Of the systems we tested, only the one that expressed the recombinant fusion protein together with the signal peptide β-lactamase (pLA71) was successful and stable *in* *vivo*. Other antigens have been expressed with the same three plasmids used in this study, but with different results. For example, the *Schistosoma mansoni* antigen Sm14 [34] and the pertussis toxin subunit S1 [39] were only successfully expressed in plasmid pLA73, which expresses the recombinant gene with the entire β-lactamase protein.

Macrophages infected with wild type BCG or rBCG produced similar amounts of NO. In the murine model, the production of NO has been shown to be critical for the control of mycobacterial growth [40]. Although NO production helps to control the progression of infection, its effects are concentration dependent. In low doses, NO acts as a signaling molecule to promote vascular integrity, mediate neurotransmission, and help regulate cellular respiration. In high concentrations, NO inhibits respiration and can cause protein and DNA damage [41]–[42]. In *M. bovis* BCG, NO seems to limit inflammatory responses, in part by down-regulating the accumulation of activated T cells [43]. We found that a significant amount of rBCG-CMX was phagocytosed, and that it can reside and survive within the macrophages (Fig. 3). Our data show that rBCG-CMX was phagocytosed in higher amounts than BCG Moreau (Figs. 3A and B). However, the induction of NO by the recombinant vaccine was similar to that induced by BCG Moreau. These data suggest that our recombinant vaccine is viable, since it has not lost its ability to induce an immune response.

After finding that rBCG-CMX was efficiently phagocytosed, we anticipated that antigen processing and presentation to naive T lymphocytes *in* *vivo* would be favored, and data from our next experiment support this idea. In cells from the lungs and spleen of rBCG-CMX vaccinated mice, stimulation with CMX induced high levels of T cells that were CD4^+^IFN-γ^+^ (Figs. 4B and C). The importance of IFN-γ in protection against TB is well established, as it induces an increase in phagocytosis and Mtb destruction, consequently reducing the bacterial load [44]. In spite of this, there is controversy about the role of IFN-γ in vaccine models. In high concentrations, IFN-γ induces apoptosis of CD4^+^ effector T lymphocytes, lowering the potential to generate memory cells [11].

Th17 cells are thought to be responsible for TB protection, as they have an early memory cell signature [45]. Our recombinant vaccine was shown to induce CMX-specific CD4^+^IL-17^+^ T cells in the spleen and lungs (Figs. 4D and E). The expression of CMX by *Mycobacterium smegmatis* (mc^2^-CMX) was also shown to induce high levels of CD4^+^IL-17^+^ T lymphocytes in the spleen and lungs [33] that directly correlated to protection. The importance of Th17 in vaccine models and in TB is controversial, but it is known that in chronic infections, such as TB, constitutive or late IL-17 production is related to the degree of interstitial inflammatory involvement and tissue lesion [46]. Instead, when produced early as is the case for vaccination, IL-17 is important for the induction of protective memory cells for TB [47], [45].

Our vaccine, rBCG-CMX, induced Th1 and Th17 immune responses that were specific to CMX. Furthermore, we demonstrated that this recombinant vaccine induced Th1 and Th17 immune responses to each of the CMX component proteins, rAg85, rMPT51, and rHspX, alone (Figs. 5A and C; Figs. 6A and C). The induction of immune responses to these proteins suggests that the construction of the CMX protein retained the immunodominant characteristics of its components. It is important to note that the use of recombinant BCG vaccines described in the literature, most of the times did not test the specific immune response to the heterologous antigens [48]–[49]. Most studies only evaluated the specific response to PPD (Purified Protein derivative) as a stimulus, and here we showed that an immune response was generated to the heterologous protein [49].

It has already been shown that rBCG expressing HspX or Ag85 complex proteins (Ag85A, Ag85B, Ag85C) induce superior protection to wild type BCG [50]–[52]. Interestingly, we found a pronounced response to stimulation with MPT51. This protein belongs to new family of non-catalytic alfa/beta hydrolases (*Fbpc*1) which act in binding the fibronectin extracellular matrix [53]. As demonstrated by another study, MPT51 effectively induces Th1 immune responses, promoting protection in mice challenged with Mtb [54]. The characteristics of these proteins were retained in CMX, which contributed to the ability of rBCG-CMX to promote important immune responses and protection.

In spleen and lung cells from mice immunized with the BCG vaccine, stimulation with rAg85 and rMPT51 induced a Th1 response, but not stimulation with HspX or CMX (Figs. 5A and B). This may be related to the poor ability of BCG to induce specific responses to certain proteins, such as HspX which is expressed in low levels by BCG [55], [50], [56]. Interestingly, despite containing the same original proteins as those composing the CMX protein, immunization with BCG Moreau did not induce a specific response against CMX. Additionally, BCG was not able to induce a Th17 immune response to any of the component recombinant proteins (Figs. 6A and B). Although it has previously been shown that Th17 responses generated by BCG vaccination induce TB infection control in non-human primates [47], we did not observe similar results with our recombinant proteins.

We found an increased number of CD4^+^ polyfunctional T cells among mice immunized with rBCG-CMX relative to the number in those who received the BCG Moreau vaccine. The recombinant vaccine induced high levels of polyfunctional T cells expressing both IL-2 and IFN-γ (Figs. 7A and B) and high levels of these cells expressing both TNF-α and IFN-γ (Figs. 7C and D). It has been demonstrated that polyfunctional cells are important for protection against intracellular bacteria, as well as viral, parasitic, and chronic bacterial infections, such as TB [57], [37]. Additionally, it has been shown that polyfunctional cells are involved in providing protection against TB [37]. Consequently, we believe that the cellular profile induced by rBCG-CMX is likely the result of our addition of CMX to BCG [32].

The ability of rBCG-CMX to induce protection against Mtb challenge showed a tendency to improve the protection conferred by BCG Moreau. Vaccination with rBCG-CMX significantly reduced the lung bacterial load of BALB/c mice (Fig. 8). Because the only difference between the vaccines were the presence of CMX, we decided to address if using a booster with rCMX would increase the immune response to CMX, and consequently the protection to Mtb. The improved protection observed (rBCG-CMX + rCMX) must be due to the extra presence of the recombinant fusion protein CMX, as only the recombinant vaccine induced a significant increase in the proliferation and migration of specific CD4^+^ T cells in the spleen and lungs (Figs. 4 and 5) of immunized mice. Like in here, not all recombinant BCG vaccines expressing fusion proteins that have been tested were able to induce superior protection when compared with BCG [58]. Thus we believe that the recombinant CMX protein, composed of Mtb immunodominant antigens (Ag85C, MPT-51, and HspX) that relate to different infection phases, added significant immunogenic properties to BCG which were crucial to the observed protection. This is the first study using limited number of animals (3–6) to demonstrate the efficacy of the fusion protein CMX. We are now setting up collaborations to test the CMX in a more appropriate guinea pig model. Other studies from our group have characterized those properties by investigating rCMX in the context of *M. smegmatis* mc^2^ 155 (mc^2^-CMX). Additionally, we observed this phenomenon with the IKE vaccine (IKE-CMX), which also induced a significant reduction in bacterial load in comparison to vaccination with IKE lacking the recombinant antigen [33]. Taken together, the data demonstrate that CMX can play an important role in the enhancement of protective immune responses induced by vaccines against Mtb [32]–[33].

Achieving the correct balance between the induction of Th1 and Th17 cells is an important goal for an effective vaccine against Mtb. While the induced IFN-γ will act on the activation of infected cells, IL-17 will regulate the resulting inflammatory response by inducing protective cells [49]. As shown in Figure 9, the lungs of mice vaccinated with rBCG-CMX had a larger preserved area of the lungs compared to the lungs of BCG Moreau immunized mice. In addition the group receiving the recombinant vaccine showed little inflammatory infiltration and very few necrotic foci and coalescent alveoli, all of which are known for being favorable areas for bacilli replication [59]–[60]. The reduced bacterial load of the lungs found in rBCG-CMX challenged mice corroborates those observations (Fig. 8). In addition, no foamy macrophages were found in the lungs of mice vaccinated with the recombinant vaccine (Fig. 9), which is important as those cells are known to be bacilli reservoirs [61]–[62].

In conclusion, the addition of the recombinant fusion protein CMX to BCG Moreau generated a recombinant vaccine with superior immunological properties. This vaccine induced a balanced IFN-γ and IL17 cytokine response from CD4^+^ T cells and was able to protect mice from Mtb.

## Supporting Information

Figure S1 **CMX expression analysis from rBCG transformed with recombinant plasmids pLA73/CMX and pMIP12/CMX.** Western blot analysis of whole cell lysates from rBCG transformants using polyclonal antibodies raised against rCMX. (A) rBCG containing pLA73/CMX or empty vector. M: molecular mass marker; CMX: purified recombinant CMX; pLA73/CMX: rBCG with plasmid pLA73/CMX; pLA73: rBCG with plasmid pLA73. (B) rBCG containing pMIP12/CMX or empty vector. M: molecular mass marker; CMX: purified recombinant CMX; pMIP12/CMX: rBCG with plasmid pMIP12/CMX; pMIP12: rBCG with plasmid pMIP12.
(TIF)

Figure S2 **Representative dot plots of TCD4^+^IFN-γ^+^ and TCD4^+^IL-17^+^ cells.** Splenic cells from non-immunized mice (Control) or mice immunized with BCG or with rBCG-CMX were stimulated with medium or one of the following recombinant proteins: rAg85, rMPT51, rHspX or rCMX. Lymphocytes were selected based on their size and granulocity and antigen specific TCD4^+^IFN-γ^+^ (A) and TCD4^+^IL-17^+^ (B) cells were analyzed based on their fluorescence.
(TIF)

## References

[1] World Health Organization (WHO) (2013) Global tuberculosis control–epidemiology, strategy, financing.

[2] Kamath AT, Fruth U, Brennan MJ, Dobbelaer R, Hubrechts P, et al (2005) New live mycobacterial vaccines: the Geneva consensus on essential steps towards clinical development. Vaccine 23: 3753–3761. 15893612 10.1016/j.vaccine.2005.03.001

[3] Calmette A (1929) Sur la vaccination preventive des enfants nouveau-nés contre tuberculose par le BCG. Ann Inst Pasteur 41: 201–232.

[4] World Health Organization (WHO) (1998) Global tuberculosis control.

[5] Partnership WST (2010) The Global Plan to Stop TB 2011–2015: Transforming the Fight- Towards Elimination of Tuberculosis.

[6] Lienhardt C, Zumla A (2005) BCG: the story continues. Lancet 366: 1414–1416. 16243075 10.1016/S0140-6736(05)67535-6

[7] Behr MA, Small PM (1997) Has BCG attenuated to impotence? Nature 389: 133–134. 10.1038/38151 9296487

[8] Zhang W, Zhang Y, Zheng H, Pan Y, Liu H, et al (2013) Genome sequencing and analysis of BCG vaccine strains. PLoS One 8: e71243. 23977002 10.1371/journal.pone.0071243 PMC3747166

[9] Soares AP, Scriba TJ, Joseph S, Harbacheuski R, Murray RA, et al (2008) Bacille Calmette–Guérin vaccination of human newborns induces T cells with complex cytokine and phenotype profiles. J Immunol 180: 3569–77. 18292584 10.4049/jimmunol.180.5.3569 PMC2842001

[10] Stenger S, Hansen DA, Teitelbaum R, Dewan P, Niazi KR, et al (1998) An antimicrobial activity of cytolytic T cells mediated by granulysin. Science 282: 121–5. 9756476 10.1126/science.282.5386.121

[11] Abebe F (2012) Is interferon-gamma the right marker for bacilli Calmette-Guérin-induced immune protection? The missing link in our understanding of tuberculosis immunology. Clin Exp Immunol 169: 213–219. 22861360 10.1111/j.1365-2249.2012.04614.x PMC3444997

[12] Mittrucker HW, Stenhoof U, Kohler A, Krause M, Lazar D, et al (2007) Poor correlation between BCG vaccination-induced T cell response and protection against tuberculosis. Proc Natl Acad A Sci USA 104: 12434–124. 10.1073/pnas.0703510104 PMC1941486 17640915

[13] Junqueira-Kipnis AP, Marques Neto LM, Kipnis A (2014) Role of Fused *Mycobacterium tuberculosis* Immunogens and Adjuvants in Modern Tuberculosis Vaccines. Front Immunol 5: 188. 24795730 10.3389/fimmu.2014.00188 PMC4005953

[14] Kaufmann SH, Lange C, Rao M, Balaji KN, Lotze M, et al (2014) Progress in tuberculosis vaccine development and host- directed therapies – a state of the art review. Lancet Respir Med 4: 301–321. 10.1016/S2213-2600(14)70033-5 24717627

[15] Hoft DF, Blazevic A, Abate G, Hanekom WA, Kaplan G, et al (2008) A new recombinant bacille Calmette-Guérin vaccine safely induces significantly enhanced tuberculosis-specific immunity in human volunteers. J Infect Dis 198: 1491–1501. 18808333 10.1086/592450 PMC2670060

[16] Deng YH, He HY, Zhang BS (2012) Evaluation of protective efficacy conferred by a recombinant *Mycobacterium bovis* BCG expressing a fusion protein of Ag85A-ESAT-6. J Microbiol Immunol Infect 25: S1684–1182. 10.1016/j.jmii.2012.11.005 23357605

[17] Tang C, Yamada H, Shibata K, Maeda N, Yoshida S, et al (2008) Efficacy of Recombinant Bacille Calmette-Guérin Vaccine Secreting Interleukin-15/Antigen 85B Fusion Protein in Providing Protection against *Mycobacterium tuberculosis* . J Infect Dis 197: 1263–1274. 18422438 10.1086/586902

[18] Farinacci M, Weber S, Kaufmann SH (2012) The recombinant tuberculosis vaccine rBCGΔureC::hly+ induces apoptotic vesicles for improved priming of CD4+ and CD8+ T cells. Vaccine 30: 7608–7614. 23088886 10.1016/j.vaccine.2012.10.031

[19] da_Costa AC, Nogueira SV, Kipnis A, Junqueira-Kipnis AP (2014) Recombinant BCG: innovations on an old vaccine. Scope of BCG strains and strategies to improve long-lasting memory. Front Immunol 5: 152. 24778634 10.3389/fimmu.2014.00152 PMC3984997

[20] Lin CW, Su IJ, Chang JR, Chen YY, Lu JJ, et al (2011) Recombinant BCG coexpressing Ag85B, CFP10, and interleukin-12 induces multifunctional Th1 and memory T cells in mice. APMIS 120: 72–82. 22151310 10.1111/j.1600-0463.2011.02815.x

[21] Gomes LH, Otto TD, Vasconcellos EA, Ferrão PM, Maia RM, et al (2011) Genome sequence of *Mycobacterium bovis* BCG Moreau, the Brazilian vaccine strain against tuberculosis. J Bacteriol 193: 5600–1. 21914899 10.1128/JB.05827-11 PMC3187452

[22] Berrêdo-Pinho M, Kalume DE, Correa PR, Gomes LH, Pereira MP, et al (2011) Proteomic profile of culture filtrate from the Brazilian vaccine strain *Mycobacterium bovis* BCG Moreau compared to *M. bovis* BCG Pasteur. BMC Microbiol 11: 80. 21507239 10.1186/1471-2180-11-80 PMC3094199

[23] Nascimento IP, Dias WO, Quintilio W, Hsu T, Jacobs WR Jr, et al (2009) Construction of an unmarked recombinant BCG expressing a pertussis antigen by auxotrophic complementation: protection against *Bordetella pertussis* challenge in neonates. Vaccine 27: 7346–51. 19782111 10.1016/j.vaccine.2009.09.043

[24] Andrade PM, Chade DC, Borra RC, Nascimento IP, Villanova Fe, et al (2010) The therapeutic potential of recombinant BCG expressing the antigen S1PT in the intravesical treatment of bladder cancer. Urol Oncol 28: 520–525. 19272796 10.1016/j.urolonc.2008.12.017

[25] Vasconcellos HL, Scaramuzzi K, Nascimento IP, Da Costa Ferreira JM Jr, Abe CM, et al (2012) Generation of recombinant bacillus Calmette-Guérin and *Mycobacterium smegmatis* expressing BfpA and intimin as vaccine vectors against enteropathogenic *Escherichia coli* . Vaccine 30: 5999–6005. 22828590 10.1016/j.vaccine.2012.05.083

[26] Clark SO, Kelly DL, Badell E, Castello-Branco LR, Aldwell F, et al (2010) Oral delivery of BCG Moreau Rio de Janeiro gives equivalent protection against tuberculosis but with reduced pathology compared to parenteral BCG Danish vaccination. Vaccine 28(43): 7109–16. 20708695 10.1016/j.vaccine.2010.07.087

[27] Yuk JM, Jo EK (2014) Host immune responses to mycobacterial antigens and their implications for the development of a vaccine to control tuberculosis. Clin Exp Vaccine Res 3: 155–167. 25003089 10.7774/cevr.2014.3.2.155 PMC4083068

[28] Achkar JM, Jenny-Avital E, Yu X, Burger S, Leibert E, et al (2010) Antibodies against immunodominant antigens of *Mycobacterium tuberculosis* in subjects with suspected tuberculosis in the United States compared by HIV status. Clin Vaccine Immunol 17: 384–392. 20071491 10.1128/CVI.00503-09 PMC2837951

[29] Rabahi MF, Junqueira-Kipnis AP, Dos Reis MC, Oelemann W, Conde MB (2007) Humoral response to HspX and GlcB to previous and recent infection by *Mycobacterium tuberculosis* . BMC Infect Dis 7: 148. 18166139 10.1186/1471-2334-7-148 PMC2241823

[30] de Araujo-Filho JA, Vasconcelos AC Jr, Martins de Sousa E, Kipnis A, Ribeiro E, et al (2008) Cellular responses to MPT-51, GlcB and ESAT-6 among MDR-TB and active tuberculosis patients in Brazil. Tuberculosis 88: 474–481 doi:10.1016/j.tube.2008.06.002 18676203 10.1016/j.tube.2008.06.002

[31] Kashyap RS, Shekhawat SD, Nayak AR, Purohit HJ, Taori GM, et al (2013) Diagnosis of tuberculosis infection based on synthetic peptides from *Mycobacterium tuberculosis* antigen 85 complex. Clin Neurol Neurosurg 115: 678–683. 22902080 10.1016/j.clineuro.2012.07.031

[32] de Sousa EM, da Costa AC, Trentini MM, de Araujo Filho JA, Kipnis A, et al (2012) Immunogenicity of a fusion protein containing immunodominant epitopes of Ag85C, MPT51, and HspX from *Mycobacterium tuberculosis* in mice and active TB infection. PLoS One 7: e47781. 23133523 10.1371/journal.pone.0047781 PMC3485045

[33] Junqueira-Kipnis AP, de Oliveira FM, Trentini MM, Tiwari S, Chen B, et al (2013) Prime-Boost with *Mycobacterium smegmatis* Recombinant Vaccine Improves Protection in Mice Infected with *Mycobacterium tuberculosis* . PLoS One 8: e78639. 24250805 10.1371/journal.pone.0078639 PMC3826754

[34] Varaldo PB, Leite CC, Dias WO, Miyaji EN, Torres FIG, et al (2004) Recombinant *Mycobacterium bovis* BCG Expressing the Sm14 Antigen of Schistosoma mansoni Protects Mice from Cercarial Challenge. Infect Immun 72: 3336–3343. 15155638 10.1128/IAI.72.6.3336-3343.2004 PMC415698

[35] Bastos RG, Borsuk S, Seixas FK, Dellagostin OA (2009) Recombinant *Mycobacterium bovis* BCG. Vaccine 27: 6495–6503. 19720367 10.1016/j.vaccine.2009.08.044

[36] Jagannath C, Lindsey DR, Dhandayuthapani S, Xu Y, Hunter RL Jr, et al (2011) Autophagy enhances the efficacy of BCG vaccine by increasing peptide presentation in mouse dendritic cells. Nat Med 15: 267–276. 10.1038/nm.1928 19252503

[37] Forbes EK, Sander C, Ronan EO, McShane H, Hill AV, et al (2008) Multifunctional, High-level cytokine-producing Th1 cells in the lung, but not spleen, correlate with protection against *Mycobacterium tuberculosis* aerosol challeng in mice.J Immunol. 181: 4955–64. 10.4049/jimmunol.181.7.4955 PMC2867031 18802099

[38] Brosch R, Gordon SV, Pym A, Eiglmeier K, Garnier T, et al (2000) Comparative genomics of the mycobacteria. Int J Med Microbiol. 290: 143–152. 10.1016/S1438-4221(00)80083-1 11045919

[39] Nascimento IP, Dias WO, Mazzantini RP, Miyaji EN, Gamberini M, et al (2000) Recombinant *Mycobacterium bovis* BCG expressing pertussis toxin subunit S1 induces protection against an intracerebral challenge with live *Bordetella pertussis* in mice. Infect Immun 68: 4877–4883. 10948100 10.1128/iai.68.9.4877-4883.2000 PMC101688

[40] Chan J, Xing Y, Magliozzo RS, Bloom BR (1992) Killing of virulent *Mycobacterium tuberculosis* by reactive nitrogen intermediates produced by activates murine macrophages. J Exp Med 175: 1111–1122. 1552282 10.1084/jem.175.4.1111 PMC2119182

[41] Xu W, Charles IG, Moncada S (2005) Nitric Oxide: orchestrating hypoxia regulation through mitochondrial respiration and the endoplasmic reticulum stress response. Cell Res 15: 63–65. 15686630 10.1038/sj.cr.7290267

[42] Pearl JE, Torrado E, Tighe M, Fountain JJ, Solache A, et al (2012) Nitric oxide inhibits the accumulation of CD4+CD44hiTbet+CD69lo T cells in mycobacterial infection. Eur J Immunol 42: 3267–3279. 22890814 10.1002/eji.201142158 PMC3664054

[43] Cooper AM, Adams LB, Dalton DK, Appelberg R, Ehlers S (2002) IFN-γ and NO in mycobacterial disease: new jobs for hands. Trends Microbiol 10: 221–226. 11973155 10.1016/s0966-842x(02)02344-2

[44] North RJ, Jung YJ (2004) Immunity to tuberculosis. Annu Rev Immunol 22: 599–623. 15032590 10.1146/annurev.immunol.22.012703.104635

[45] Muranski P, Borman ZA, Kerkar SP, Klebanoff CA, Ji Y, et al (2011) Th17 Cells Are Long Lived and Retain a Stem Cell-like Molecular Signature. Immunity 35: 972–985. 22177921 10.1016/j.immuni.2011.09.019 PMC3246082

[46] Cruz A, Fraga AG, Fountains JJ, Rangel-Moreno J, Torrado E, et al (2010) Pathological role of interleukin 17 in mice subjected to repeated BCG vaccination after infection with *Mycobacterium tuberculosis* . J Exp Med 207: 1609–1616. 20624887 10.1084/jem.20100265 PMC2916141

[47] Wareham AS, Tree JA, Marsh PD, Butcher PD, Dennis M, et al (2014) Evidence for a role for interleukin-17, Th17 cells and homeostasis in protective immunity against tuberculosis in cynomolgus macaques. PloS One 9: e88149. 24505407 10.1371/journal.pone.0088149 PMC3913765

[48] Tullius MV, Harth G, Maslesa-Galic S, Dillon BJ, Horwitz MA (2008) A replication-limited recombinant *Mycobacterium bovis* BCG vaccine against tuberculosis designed for human immuno deficiency virus-positive persons is safer and more efficacious than BCG. Infect Immun 76: 5200–14. 18725418 10.1128/IAI.00434-08 PMC2573348

[49] Desel C, Dorhoi A, Bandermann S, Grode L, Eisele B, et al (2011) Recombinant BCGΔureC::hly Induces Superior Protection over Parental BCG by Stimulating a Balanced Combination of Type 1 and Type 17 Cytokine Responses. J Infect Dis 204: 1573–1584. 21933877 10.1093/infdis/jir592 PMC3192191

[50] Shi C, Chen L, Chen Z, Zhang Y, Zhou Z, et al (2010) Enhanced protection against tuberculosis by vaccination with recombinant BCG over-expressing HspX protein. Vaccine 28: 5237–5244. 20538090 10.1016/j.vaccine.2010.05.063

[51] Wang C, Fu R, Chen Z, Tan K, Chen L, et al (2012) Immunogenicity and protective efficacy of a novel recombinant BCG strain over expressing antigens Ag85A e Ag85B. Clin. Dev. Immunol. 2012: 1–9. 10.1155/2012/563838 PMC3337592 22570667

[52] Jain R, Dey B, Dhar N, Rao V, Singh R, et al (2008) Enhanced and enduring protection against tuberculosis by recombinant BCG-Ag85C and its association with modulation of cytokine profile in lung. PLoS One 3: e3869. 19052643 10.1371/journal.pone.0003869 PMC2586085

[53] Wilson RA, Maughan WN, Kremer L, Besra GS, Fütterer K (2004) The structure of *Mycobacterium tuberculosis* MPT51 (FbpC1) defines a new family of non-catalytic alpha/beta hydrolases. J Mol Biol 9: 519–530. 10.1016/j.jmb.2003.11.001 14672660

[54] Silva BD, da Silva EB, do Nascimento IP, Dos Reis MC, Kipnis A, et al (2009) MPT-51/CpG DNA vaccine protects mice against *Mycobacterium tuberculosis* . Vaccine 27: 4402–4407. 19500525 10.1016/j.vaccine.2009.05.049

[55] Geluk A, Lin MY, van Meijgaarden KE, Leyten EM, Franken KL, et al (2007) T-cell recognition of the HspX protein of *Mycobacterium tuberculosis* correlates with latent *M. tuberculosis* infection but not with *M. bovis* BCG vaccination. Infect Immun 75: 2914–2921. 17387166 10.1128/IAI.01990-06 PMC1932904

[56] Spratt JM, Britton WJ, Triccas JA (2010) In vivo persistence and protective efficacy of the bacille Calmette Guerin vaccine overexpressing the HspX latency antigen. Bioeng Bugs 1: 61–65. 21327127 10.4161/bbug.1.1.10027 PMC3035152

[57] Maroof A, Yorgensen YM, Li Y, Evans JT (2014) Intranasal vaccination promotes detrimental Th17-mediated immunity against influenza infection. PLoS Pathog 10: e1003875. 24465206 10.1371/journal.ppat.1003875 PMC3900655

[58] Deng YH, He HY, Zang BS (2014) Evaluation of protective efficacy conferred by a recombinant *Mycobacterium bovis* BCG expressing a fusion protein Of Ag85A-ESAT-6. J Microbiol Immunol Infect 47: 48–56. 23357605 10.1016/j.jmii.2012.11.005

[59] Ulrichs T, Kosmiadi GA, Jorg S, Pradl L, Titukhina M, et al (2005) Differential organization of the local immune response in patients with active cavitary tuberculosis or with nonprogressive tuberculoma. J Infect Dis192: 89–97. 10.1086/430621 15942898

[60] Lenaerts AJ, Hoff D, Aly S, Ehlers S, Andries K, et al (2007) Mycobacteria in a guinea pig model of tuberculosis revealed by r207910. Antimicrob Agents Chemother 51: 3338–3345. 17517834 10.1128/AAC.00276-07 PMC2043239

[61] Russell DG, Cardona PJ, Kim MJ, Allain S, Altare F (2009) Foamy macrophages and the progression of the human tuberculous granuloma. Nature Immunol 10: 943–948. 19692995 10.1038/ni.1781 PMC2759071

[62] Huynh KK, Joshi SA, Brown EJ (2011) A delicate dance: host response to mycobacteria. Current Opinion in Immunology 23: 464–472. 21726990 10.1016/j.coi.2011.06.002

