# Correction: A New Recombinant BCG Vaccine Induces Specific Th17 and Th1 Effector Cells with Higher Protective Efficacy against Tuberculosis

**DOI:** 10.1371/journal.pone.0116033

**Published:** 2014-12-15

**Authors:** The PLOS ONE Staff

Figure 8 is incorrect. Please view the correct Figure 8 here.

**Figure 8:**
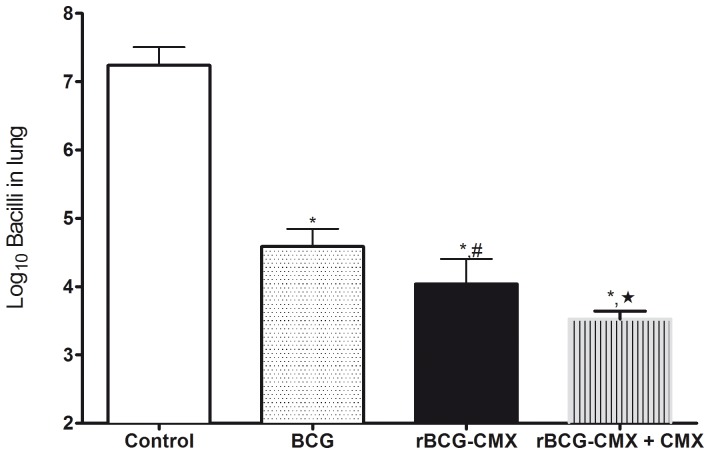
Bacterial load in the lungs of BALB/c mice 45 days after *Mycobacterium tuberculosis* challenge. Ninety days after immunization, three mice from each group (control, BCG and rBCG-CMX) were challenged with 10^5^ CFU of *Mycobacterium tuberculosis* H37Rv intravenously into the orbital sinus plexus. One additional group of animals received a booster of rCMX/CPG DNA, 30 days after rBCG-CMX vaccination and challenged with Mtb 30 days post the immunization (rBCG-CMX+CMX). Forty-five days after challenge, mice were euthanized and the anterior and mediastinal right lung lobes were collected, homogenized, and plated on Middlebrook 7H11agar supplemented with OADC to determine the bacterial load by counting the number of CFU. * Significant differences between infected (control) and vaccinated groups. # Significant differences between rBCG-CMX and rBCG-CMX+CMX groups. | Significant differences between rBCG-CMX and BCG groups analyzed by *t* test (p<0.05).
