# Supplementary figures and images for: A New Recombinant BCG Vaccine Induces Specific Th17 and Th1 Effector Cells with Higher Protective Efficacy against Tuberculosis
Source: PLoS One. 2014 Nov 14;9(11):e112848. doi: 10.1371/journal.pone.0112848 (PMC4232451; doi:10.1371/journal.pone.0112848)

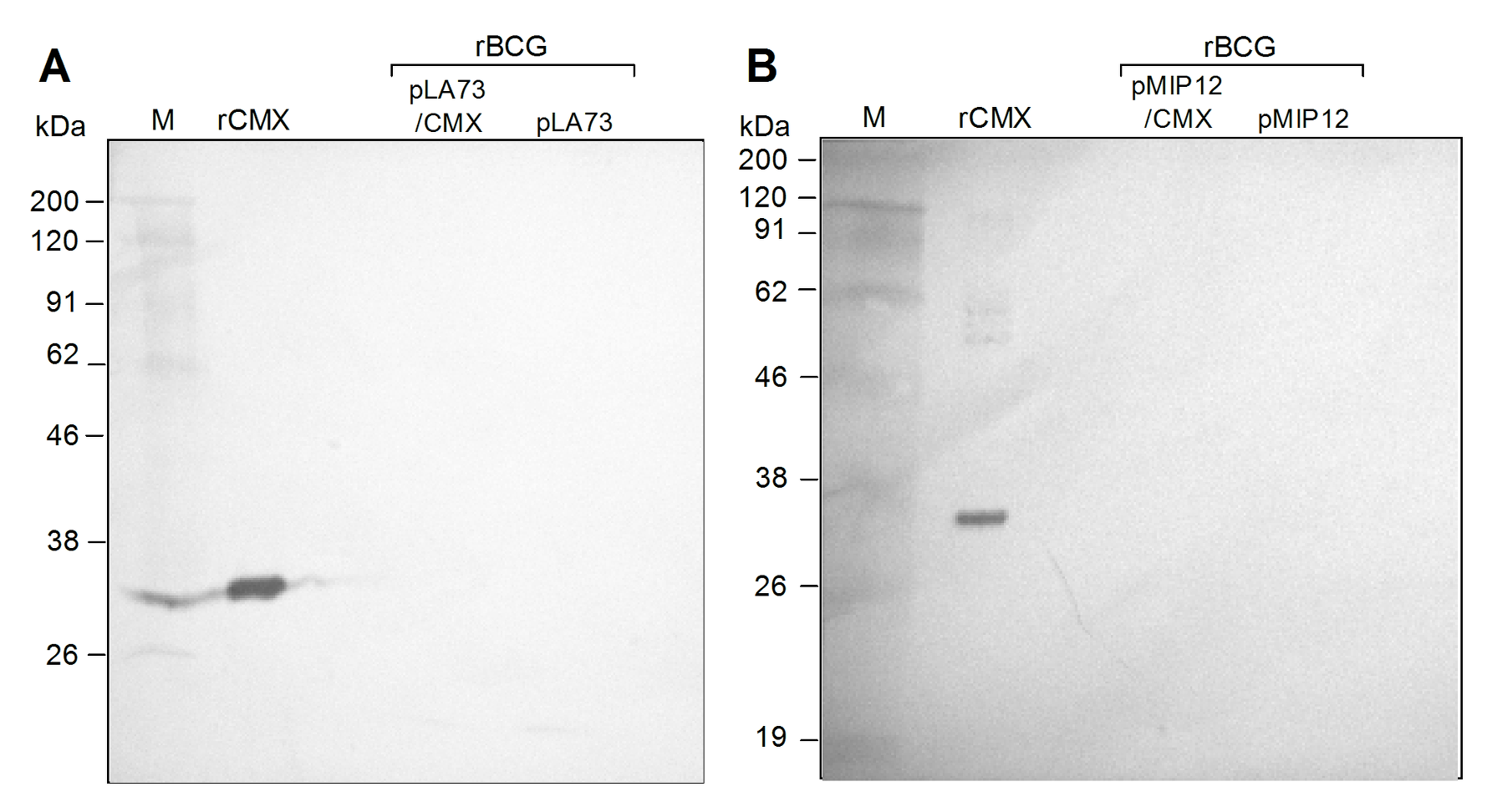

Supplement: Figure S1 — CMX expression analysis from rBCG transformed with recombinant plasmids pLA73/CMX and pMIP12/CMX. Western blot analysis of whole cell lysates from rBCG transformants using polyclonal antibodies raised against rCMX. (A) rBCG containing pLA73/CMX or empty vector. M: molecular mass marker; CMX: purified recombinant CMX; pLA73/CMX: rBCG with plasmid pLA73/CMX; pLA73: rBCG with plasmid pLA73. (B) rBCG containing pMIP12/CMX or empty vector. M: molecular mass marker; CMX: purified recombinant CMX; pMIP12/CMX: rBCG with plasmid pMIP12/CMX; pMIP12: rBCG with plasmid pMIP12. (TIF) [file pone.0112848.s001.tif]

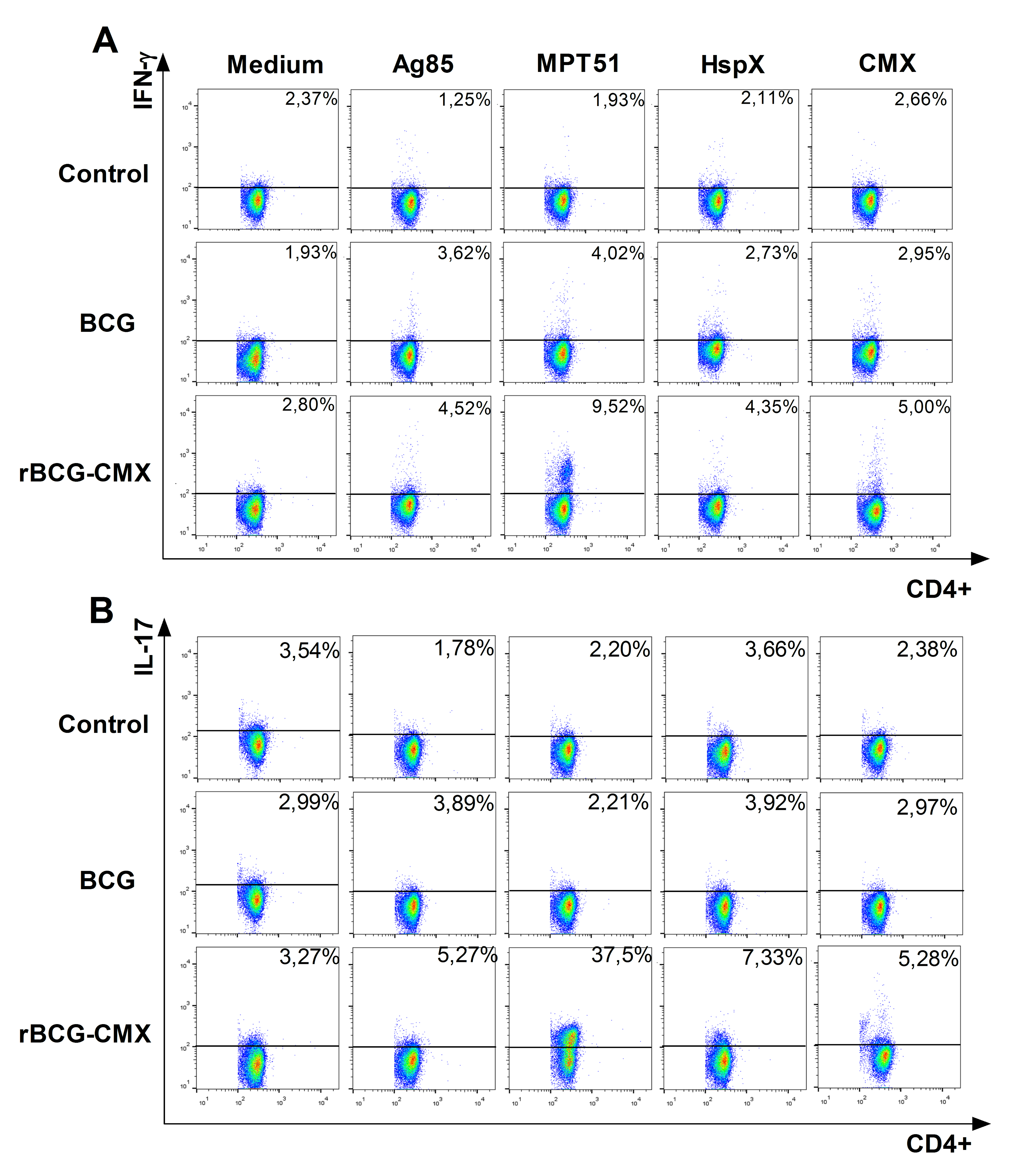

Supplement: Figure S2 — Representative dot plots of TCD4+IFN-γ+ and TCD4+IL-17+ cells. Splenic cells from non-immunized mice (Control) or mice immunized with BCG or with rBCG-CMX were stimulated with medium or one of the following recombinant proteins: rAg85, rMPT51, rHspX or rCMX. Lymphocytes were selected based on their size and granulocity and antigen specific TCD4+IFN-γ+ (A) and TCD4+IL-17+ (B) cells were analyzed based on their fluorescence. (TIF) [file pone.0112848.s002.tif]
